# Effect of Obesity on Mortality in Pulmonary Hypertension—A Systematic Review and Meta-Analysis

**DOI:** 10.3390/jcdd10100419

**Published:** 2023-10-06

**Authors:** Raju Reddy, Saminder Singh Kalra, Bashar Alzghoul, Akram Khan, Yazan Zayed

**Affiliations:** 1 Division of Pulmonary and Critical Care Medicine, University of Texas at Austin, Austin, TX 78712, USA; 2 Division of Pulmonary, Critical Care and Sleep Medicine, University of Florida, Gainesville, FL 32611, USA; saminder.kalra@medicine.ufl.edu (S.S.K.); bashar.alzghoul@medicine.ufl.edu (B.A.); yz.alzayed@gmail.com (Y.Z.); 3 Division of Pulmonary, Allergy and Critical Care Medicine, Oregon Health and Science University, Portland, OR 97239, USA; khana@ohsu.edu

**Keywords:** obesity, pulmonary hypertension, mortality, body mass index

## Abstract

Obesity is reported to have a protective effect on mortality in pulmonary hypertension (PH), a phenomenon known as obesity paradox. However, the data are conflicting, with some studies showing decreased mortality while other studies found no effect of obesity on mortality. Therefore, we performed a systematic review and meta-analysis to examine whether there is an association between obesity and mortality in PH. Only patients with PH diagnosed by right heart catheterization were included. We also performed a sub-group analysis of subjects with pre-capillary PH only. A total of six studies met the inclusion criteria, with a sample size of 13,987 patients. Obese subjects had lower mortality compared to non-obese subjects in the combined pre- and post-capillary PH group (hazard ratio 0.79, 95% CI 0.66–0.95, *p* = 0.01). While obesity was associated with reduction in mortality in the pre-capillary PH group (hazard ratio 0.77, 95% CI 0.60 to 0.98, *p* = 0.03), this was not uniform across all studies.

## 1. Introduction

Approximately one third of adult Americans suffer from obesity [1]. The impact of obesity on cardiovascular outcomes and mortality has been examined in several studies. Obese patients are more likely to develop coronary artery disease requiring revascularization and are more likely to be diagnosed with left heart failure as well [2,3]. However, obese patients with left-sided heart failure have higher survival rates compared to their non-obese counterparts [4]. In addition, obese patients also have lower rates of hospitalization [4]. This phenomenon is known as the obesity paradox [5,6,7]. Similar findings have been reported in patients with pre-capillary pulmonary hypertension (PH) where obese patients have higher survival rates [8,9,10,11]. However, not all studies examining this relationship have found a similar association [12,13]. Furthermore, amongst patients with left heart failure, not all classes of obesity were associated with lower mortality. For instance, class 3 obese (body mass index (BMI) ≥ 35 kg/m^2^) heart failure patients have significantly higher mortality than normal BMI patients. Thus, the interplay between obesity and clinical outcomes is complex and not a monotonic relationship. Whether a similar pattern is present in patients with pre-capillary PH remains unknown. To examine this association, we sought to examine the effect of obesity on mortality amongst PH subjects by performing a systematic review and meta-analysis using the Preferred Reporting Items for Systematic Reviews and Meta-Analyses Protocols (PRISMA) 2015 Statement guidelines [14].

## 2. Methods

### 2.1. Study Design and Study Selection

A comprehensive literature search utilizing major electronic databases including PubMed and Cochrane library was performed using the MeSH term “obesity AND pulmonary hypertension”. We first screened articles by their abstracts and titles. We then reviewed eligible titles further by reviewing the full texts before their final inclusion or exclusion based on the predefined criteria. In addition, we assessed the references of relevant articles, and a manual web search was also performed. Two reviewers independently and separately conducted the literature search (R.R and Y.Z.). Any discrepancy was solved by a consensus with a third reviewer (S.K.).

### 2.2. Inclusion and Exclusion Criteria

We included all studies that evaluated the effect of obesity (defined as BMI ≥ 30 kg/m^2^) in patients with pulmonary hypertension in comparison to non-obese patients (BMI < 30 kg/m^2^) or normal weight patients (BMI 18.5–25 kg/m^2^). Retrospective studies and prospective studies were included. Only studies with hemodynamic data obtained by right heart catheterization and reported mortality outcomes were included. We excluded studies that evaluated the effect of obesity exclusively in patients with group 2 pulmonary hypertension. We excluded case reports, reviews, and meta-analyses.

### 2.3. Outcome Definitions

The main outcome was the association of obesity on the mortality of patients with pulmonary hypertension from the time since diagnosis reported as a hazards ratio (HR). We also examined mortality and hemodynamic parameters such as mean pulmonary arterial pressure, pulmonary capillary wedge pressure, and cardiac output according to BMI sub-groups (<18.5 kg/m^2^, 18.5–24.9 kg/m^2^, 25–34.9 kg/m^2^, ≥35 kg/m^2^). Lastly, other outcomes including hospitalizations and quality of life (QOL) scores between the obese and non-obese groups were obtained from the included studies. As BMI sub-group mortality, hemodynamic parameters, hospitalizations, and QOL scores were reported inconsistently between studies; these data could not be pooled for meta-analysis.

### 2.4. Data Extraction

Three reviewers (R.R., S.K., and Y.Z.) independently and separately extracted the outcomes of interest. In addition, we extracted the baseline characteristics of the patient population and the characteristics of the studies.

### 2.5. Quality Assessment

We assessed the quality of included studies using the Newcastle–Ottawa Scale which examined the included studies based on three broad categories, including the selection of the study groups; comparability of the groups; and ascertainment of the outcomes of interest [15]. Two reviewers (Y.Z. and R.R.) performed the quality assessment separately and independently.

### 2.6. Statistical Analysis

We used the generic inverse variance to calculate the pooled HR of mortality and its 95% corresponding confidence interval using a random effects model comparing obese (BMI ≥ 30 kg/m^2^) to non-obese (BMI < 30 kg/m^2^) or normal weight patients (BMI 18.5–25 kg/m^2^). When HR was not reported, we excluded the study from the final analysis. We performed the primary analysis by including all the studies that included patients with mixed etiology for PH and a sub-group analysis of patients with pre-capillary PH only. Since the majority (65%) of patients in the pre-capillary PH group were from one study [11], we also performed a sensitivity analysis by excluding this study [11] and then repeated the analysis.

## 3. Results

**Figure 1:**
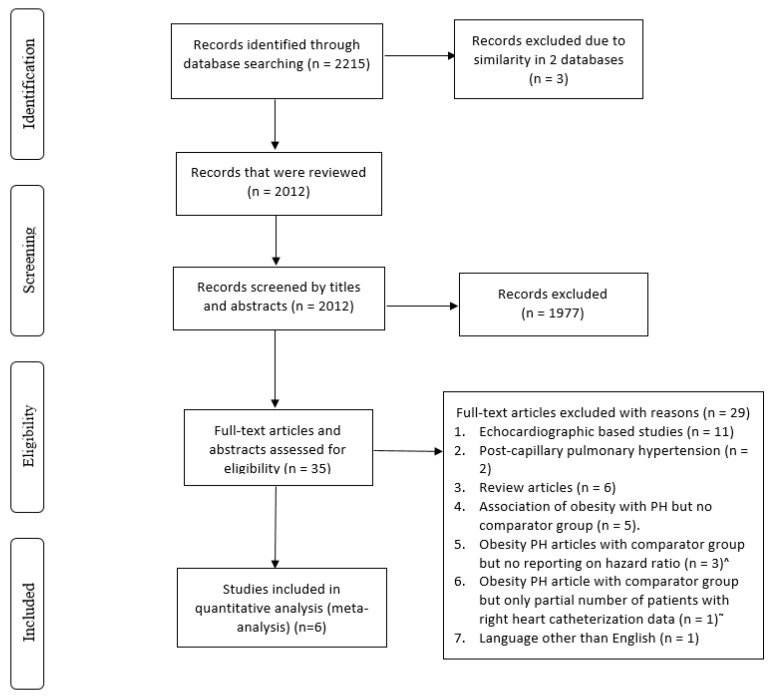
Flow diagram of study selection. ^^^ Excluded articles: [10,13,18]. ^~^ Excluded article: [19].

Study sample: After the final review by three authors (R.R., S.K., and Y.Z.), six studies met the inclusion criteria (Figure 1 and Table 1). All six studies were prospective observational studies. Two studies included subjects with pre-capillary PH, post-capillary PH, and combined pre- and post-capillary PH [11,16], while the other four studies included only subjects with pre-capillary PH [8,9,12,17] (Table 1). Four studies defined PH as mean pulmonary artery pressure (MPAP) ≥ 25 mm Hg, pulmonary capillary wedge pressure (PCWP) ≤ 15 mm Hg, and pulmonary vascular resistance (PVR) ≥ 3 WU [9,10,12,17], and two studies used a different definition [8,11] (Table 1). Follow-up duration varied between studies. Two were single-center studies [11,16], and the remainder were prospective registry-based studies [8,9,12,17] (Table 1). The study participants were from the United States [9,11,17], France [12], Israel [16], and Australia and New Zealand [8]. All studies classified obese individuals as those with BMI ≥ 30 kg/m^2^. Five studies [8,11,12,16,17] compared obese (BMI ≥ 30 kg/m^2^) versus non-obese subjects (BMI < 30 kg/m^2^), while one study [9] compared obese (BMI ≥ 30 kg/m^2^) versus normal weight subjects (BMI 18–24.9 kg/m^2^). The quality assessment of the included studies is explained in Table 2. Three additional studies of group 1 PH patients examined the association between obesity and mortality, but no comparator groups were present [10,13,18] and were thus not included in the analysis. The final analysis totaled 13,987 patients; 5187 (37.1%) were obese subjects defined as BMI ≥ 30 kg/m^2^, and 8800 (62.9%) were non-obese or normal weight subjects. In total, 5847 (41.8%) subjects were female.

**Figure 2:**
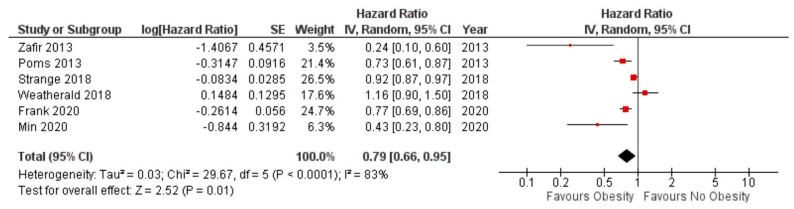
Forest plot for mortality amongst obese versus non−obese subjects in combined pre− and post−capillary pulmonary hypertension.

**Figure 3:**
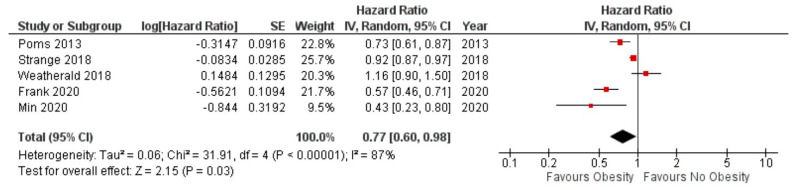
Forest plot for mortality amongst obese versus non−obese subjects in pre−capillary pu−monary hypertension only.

**Figure 4:**
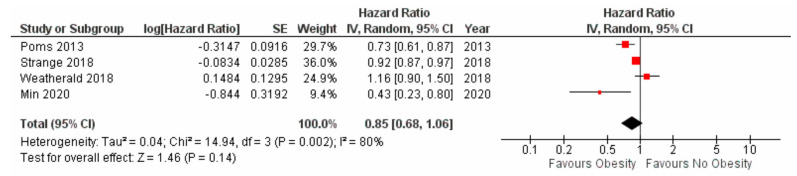
Forest plot for mortality amongst obese versus non−obese subjects with pre−capillary pulmonary hypertension (exclusion of reference [11]).

Mortality: Obese subjects had significantly reduced mortality when compared to non-obese subjects in the mixed PH patient population (hazard ratio 0.79, 95% CI 0.66–0.95, *p* = 0.01) (Figure 2) as well as in subjects with pre-capillary PH (hazard ratio 0.77 95% CI 0.60 to 0.98, *p* = 0.03) (Figure 3). As the majority (65%) of the patients were obtained from one study, we performed a sensitivity analysis by excluding the study by Frank et al. [11]. The remaining patients totaled 5047 patients. In this sub-group analysis, we did not have sufficient information to conclude that obesity was associated with a reduction in mortality [hazard ratio 0.85, 95% CI 0.68 to 1.06), *p* = 0.14)] (Figure 4).

Obesity sub-group analysis of mortality, hemodynamics, hospitalizations, and quality of life scores. Only two studies [10,12] examined the relationship between obesity sub-class and mortality. Both found a U-shaped mortality curve with higher mortality rates in underweight (BMI < 18.5 kg/m^2^) and class 3 obesity (BMI ≥ 35 kg/m^2^) compared to normal (BMI 18.5–24.9 kg/m^2^) and overweight subjects (BMI 25–34.9 kg/m^2^). Obesity class-based sub-group hemodynamics were reported by two groups [11,12]. In the largest study of 8940 patients by Frank et al., higher MPAP (*p* < 0.0001) and higher PCWP (*p* < 0.0001) were noted with increasing obesity sub-class [11]. Similarly, the studies by Weatherald et al. reported statistically significant higher PCWP with increasing BMI (*p* < 0.001) [12]. Cardiac output was reported in only one study [12]. Obese patients had higher cardiac output compared to their non-obese counterparts (*p* < 0.001). The study by Min et al. also reported higher cardiac output in the obese group, but no statistical comparison was made with the overweight and normal weight groups [9]. PVR was lower in higher obesity sub-class (*p* < 0.001) in the study by Weatherald et al. [12]. Similarly, Min et al. also reported lower PVR in the obese group compared to overweight and normal weight patients, but no statistical analysis was performed [9]. Finally, Min et al. found higher rates of hospitalization amongst overweight and obese patients compared to normal weight patients, but it was not statistically significant (incidence rate ratio 1.33; 95% CI, 0.93–1.89; *p* = 0.09) [9]. However, obese patients and overweight patients had lower quality of life scores (higher e10 scores indicating worse quality of life) compared to normal weight patients (*p* = 0.01 and *p* < 0.001, respectively) [9].

## 4. Discussion

Our study showed that obesity is present in 37.1% of subjects with PH and was associated with a significant reduction in mortality amongst subjects with both pre-capillary PH as well as combined groups of pre- and post-capillary PH when compared with non-obese subjects. Amongst studies that reported hemodynamics, obese and overweight patients had higher MPAP, higher PCWP, and higher cardiac output.

Our study findings were consistent with a study by Jiang et al., who also explored the link between obesity and mortality in patients with pulmonary hypertension [20]. In this meta-analysis consisting of 127,215 participants, the authors found that for every 5 unit increase in BMI, a risk reduction (RR 0.83 (95% CI 0.77–0.89)) in mortality was noted, with the lowest risk in those with BMI 32–38 kg/m^2^. While the large number of participants was impressive, 110,495 participants were from one study (Trammell et al.) [19]. The study by Trammell et al. used ICD9 codes 416.0, 416.2, 416.8, and 416.9, which correspond to chronic pulmonary heart disease, chronic pulmonary embolism, other chronic pulmonary heart diseases, and chronic pulmonary heart disease unspecified, respectively. Therefore, over half of the subjects (*n* = 63,602, 57.6%) were classified as “Multiple causes of PH”. While conducting research using ICD diagnosis is convenient, positive predictive value for chronic conditions may range from 39 to 100% [21]. As such, we believe that inclusion in this study by Jiang et al. is a major limitation. Our study only included patients with PH diagnosed via right heart catheterization.

The mechanism of the protective effect of obesity on mortality in cardiovascular disease is unknown, but a few hypotheses have been explored. Adiponectin, a hormone derived from adipocytes, is thought to play a protective role in cardiovascular diseases by decreasing inflammatory cytokines such as tumor necrosis factor α levels and the inhibition of NF-κβ activity [22]. In a study by Kumada et al., low levels of adiponectin were associated with the development of coronary artery disease and myocardial infarction in humans [23]. Similarly, in animal models of PH, it has been shown that adiponectin is associated with reduced smooth muscle cell proliferation and decreasing levels of inflammatory cytokines [22]. Thus, patients who are normal or underweight may lose the protective effect of adiponectin. In addition, adipose tissue secretes apelin, an adipokine, which is known to promote vasodilation by activating nitric oxide synthase [24]. Whether the vasodilatory effect of apelin exists in obese PH patients remains unknown is an area for further research.

While the association between obesity and reduced mortality is interesting, it should be interpreted with caution for several reasons. First, the pathologic process in obese PH patients and their non-obese counterparts may be different. In three of the included studies [9,11,17], obese patients tended to have higher PCWP, suggesting that they may have occult left ventricular diastolic dysfunction despite meeting the criteria for pre-capillary PH at the time of right heart catheterization, a finding present in other studies as well [25,26]. In the study consisting of a 1043-person mixed PH patient population by Thayer et al., the authors utilized mendelian randomization to examine the effect of BMI on hemodynamics [26]. They found that BMI genetics were associated with pulmonary venous hypertension rather than pulmonary arterial remodeling and thus concluded that obesity was not associated with the development of pre-capillary disease. Second, obese patients in the same studies had a higher cardiac output and lower PVR [9,12], which could also contribute to improved outcomes in these patients [27]. Third, rather than obesity conferring a protective effect, it is also possible that a low BMI may simply be a manifestation of declining health due to right ventricular dysfunction. This decline in right ventricular failure may manifest as cardiac cachexia, characterized by the congestion of hepatic and splanchnic beds, intestinal dysmotility, and protein malabsorption [28]. In two of the included studies, patients with lower BMIs had a lower cardiac index [9,12]. This association of low BMI and higher mortality was also reported in left heart failure [4,29]. Fourth, BMI does not capture body composition accurately. The implication that a higher BMI is related to higher fat mass is not necessarily true. Thus, a better predictor of mortality could be the total fat mass or the pattern of the distribution of fat (central vs. peripheral). In a study of heart failure patients by De Schutter et al., a J-shaped mortality curve was seen where patients with a BMI < 18.9 kg/m^2^ and BMI > 35 kg/m^2^ had higher mortality compared to the overweight BMI 25–35 kg/m^2^ group [29]. On further analysis, it was found that having a higher lean body mass despite being overweight conferred a protective effect, and that a higher body fat percentage in the obese population was detrimental. A similar J-shaped mortality curve was also observed in patients with pre-capillary PH as well [10,12]. While these studies did not examine body mass composition, it is possible that the morbidly obese PH patients who had a higher mortality had a higher fat mass percentage. Finally, lead time bias is possible, where obese patients may have been diagnosed earlier with PH compared to their non-obese counterparts as they may have symptoms earlier, thus leading to longer survival times [26]. Thus, these results should be interpreted cautiously, and future studies comparing outcomes (including mortality) based on fat distribution or the total fat mass may be more useful rather than BMI alone.

Our analysis is limited by its heterogeneity. We believe the heterogeneity in our study is largely driven by the clinical heterogeneity of the included studies and could be explained with the following reasons. First, there was considerable variation between studies regarding the definition of pre-capillary PH. While the majority of the studies defined PH as MPAP ≥ 25 mm Hg, PCWP ≤ 15 mm Hg, and PVR ≥ 3 WU, two studies [8,11] used different definitions. Second, there were significant differences in the study population between studies. For example, the studies by Strange et al. and Weatherald et al. [8,12] included patients with incident, drug-induced, and heritable pre-capillary PH only, while the remainder of the studies included all etiologies of group 1 PH. We do not believe this contributed significantly to the study’s heterogeneity as the most common cause of group 1 PH is idiopathic, and thus any variation in the study population is minor. Third, the variables used to adjust for confounding in studies differed, which could have affected the HR estimates and the confidence intervals obtained when studying the impact of obesity in PH patients. For example, some studies [9,12,16] controlled for comorbidities, right ventricular afterload such as PVR, and cardiac function, but this was not consistent across studies. Unfortunately, we were not able to perform a meta-regression analysis based on these variables and other study-level covariates to examine the source of heterogeneity because of the different format of reporting these data by the included studies.

In summary, we found that obese PH patients had lower mortality compared to non-obese patients. However, this finding must be interpreted with caution as BMI does not necessarily equate to fat mass, which may be a better predictor of mortality. Thus, there may be an ‘obesity paradox’ in PH, a noteworthy finding that warrants further studies with the control of other confounding variables that might contribute to these findings. Future studies examining body fat distribution in obese and non-obese patients with PH may help understand the association between BMI and mortality in this group. With the precise causal effects of obesity in PH being unknown, future registry-based studies could increase the long-term follow-up of patients and closely track body composition to determine the effect of obesity more precisely in PH.

## Figures and Tables

**Table 1:** Summary of clinical details of included studies.

Study Name (First Author and Year)	Study Design	Obesity Definition	Pulmonary Hypertension Definition	Study Groups and Patient Numbers	Mortality Hazard Ratio (95% CI*)	Follow-Up Duration	Limitations	Comments
Zafir 2013	Single-center, prospective	BMI ≥ 30 kg/m^2^	Pre-capillary PH defined as MPAP > 25 mm Hg, PCWP ≤ 15 mm Hg, and PVR ≥ 3 WU; post-capillary PH defined as MPAP > 25 mm Hg, PCWP > 15 mm Hg, PVR ≥ 3 WU.	Total = 105; BMI < 30, *n* = 62; BMI > 30, *n* = 43	BMI > 30, HR 0.2 (0.1–0.6, *p* = 0.004)	19 ± 13 months	Small sample size; single-center; inclusion of pre-capillary and post-capillary PH; no sub-group analysis of survival by class I–III obesity.	Higher mortality in low-BMI groups in both pre- and post-capillary PH; adjusted for functional class, pericardial effusion, BMI, moderate–severe TRV, right ventricular dysfunction, systolic blood pressure, MPAP, PVR, RAP, hemoglobin level.
Poms 2013	Multicenter, prospective (REVEAL registry)	BMI ≥ 30 kg/m^2^	Pre-capillary PH defined as MPAP > 25 mm Hg at rest or more than 30 mm Hg with exercise, PCWP ≤ 15 mm Hg, and PVR ≥ 3 WU.	Total = 2959; BMI ≥ 30, *n* = 956	BMI ≥ 30, HR 0.73 (0.61–0.86, *p* < 0.001)	3 years	Obesity defined by BMI and not by adipose distribution pattern; no sub-group analysis of survival by class I–III obesity.	Adjusted for hypertension, clinical depression, DM, obesity, COPD, OSA, and thyroid disease; amongst obese patients, functional class III/IV was associated with increased HR 1.37 (1.14–1.65).
Strange 2018	Multicenter, Prospective (PHSANZ registry)	BMI ≥ 30 kg/m^2^	Pre-capillary PH defined as MPAP ≥ 25 mm Hg and PCWP ≤ 15 mm Hg	Total = 220; obese BMI ≥ 30, *n* = 75	BMI ≥ 30, HR 0.91 (0.87–0.97), *p* = 0.008)	26 months	Data limited to idiopathic, heritable, and drug-induced PH; obesity defined by BMI and not by adipose distribution pattern; no sub-group analysis of survival by class I–III obesity.	Adjusted for sex and six-minute walk distance.
Weathearald 2018	Multicenter, Prospective (FHPR)	BMI ≥ 30 kg/m^2^; morbid obesity BMI ≥35 kg/m^2^	Pre-capillary PH defined as MPAP ≥ 25 mm Hg, PCWP ≤ 15 mm Hg, and PVR ≥ 3 WU	Total = 1255; BMI < 30, *n* = 874; BMI ≥ 30, *n* = 381	BMI < 18.5, *n* = 57, HR 1.76 (0.97–3.19, *p* = 0.06); BMI 18.5–24.9, *n* = 416, HR 1; BMI 25–29.9, *n* = 401, HR 0.85 (0.64–1.12, *p* = 0.24); BMI 30–34.9, *n* = 240, HR 0.98 (0.71–1.36, *p* = 0.92); BMI > 35, *n* = 141, HR 1.42 (0.95–2.14, *p* = 0.09); Obesity HR 1.16 (0.9–1.5, *p* = 0.26)	Average of 3 years	Data limited to idiopathic, heritable, and drug-induced PH; serial BMI not measured over time; obesity defined by BMI and not by adipose distribution pattern.	Adjusted for age, sex, etiology of pulmonary arterial hypertension, systemic hypertension, DM, smoking, New York Heart Association functional class, RAP, MPAP, and CI. Age < 65, morbidly obese patients had higher mortality HR 3 (1.56–5.79, *p* = 0.001). Among patients > 65 years, there was no significant association between higher BMI and mortality after adjusting for potential confounders, but there was an increase in mortality for patients who were underweight.
Min 2020	Multicenter, prospective (PHAR)	BMI ≥ 30 kg/m^2^	Pre-capillary PH defined as MPAP ≥ 25 mm Hg, PCWP ≤ 15 mm Hg and PVR ≥ 3 WU	Total = 767; BMI 18.5–24.9, *n* = 204; BMI 25–29.9, *n* = 259; BMI > 30, *n* = 304	BMI ≥ 30, HR 0.43 (0.23–0.78, *p* = 0.006); BMI 25–29.9, HR 0.48 (0.25–0.89, *p* = 0.002)	Median 512–557 days	Underweight patients were excluded; 7% of patients were lost to follow-up; obesity defined by BMI and not by adipose distribution pattern; comorbidities associated with obesity such as OSA and DM were not available; no sub-group analysis of survival by class I–III obesity.	Adjusted for age, sex, race/ethnicity, PAH etiology, cardiac index, RAP, combination therapy, parenteral prostacyclin analog use, use of supplemental oxygen, and lung transplantation; overweight subjects and obese subjects had worse HQROL compared with normal-weight patients (*p* = 0.001 and *p* = 0.01, respectively).
Frank 2020	Single-center, prospective	BMI ≥ 30 kg/m^2^	PH defined as MPAP ≥ 20 mm Hg; pre-capillary PH defined as MPAP ≥ 20 mm Hg, PCWP ≤ 15 mm Hg and TPG ≥ 12 mm Hg	Total = 8940; BMI ≥ 30, *n* = 3428	Obesity PH BMI ≥ 30, HR 0.77 (0.69–0.85, *p* < 0.001); obesity pre-capillary PH BMI ≥ 30, HR 0.57, (0.47–0.70, *p* < 0.001)	5.5 years	Referral bias and confounding by indication; single-center; obesity defined by BMI and not by adipose distribution pattern.	Multivariable model adjusted for age, sex, heart rate, hypertension, DM, OSA, chronic kidney disease, previous myocardial infarction, and heart failure.

BMI = body mass index; CI = cardiac index; CI* = confidence interval; COPD = chronic obstructive pulmonary disease; DM = diabetes mellitus; FHPR = French Pulmonary Hypertension Registry; HR = hazard ratio; HRQoL = health-related quality of life; mm Hg = millimeters of mercury; MPAP = mean pulmonary arterial pressure; OSA = obstructive sleep apnea; PCWP = pulmonary capillary wedge pressure; PH = pulmonary hypertension; PHANZ = Pulmonary Hypertension Society of Australia and New Zealand; PHAR = Pulmonary Hypertension Association Registry; PVR = pulmonary vascular resistance; RAP = right atrial pressure; REVEAL = Registry to Evaluate Early and Long-term Pulmonary Arterial Hypertension; TRV = tricuspid regurgitant velocity; WU = wood units.

**Table 2:** Quality assessment of studies.

Study (Author and Year)		Selection		Comparability		Outcome		AHRQ Standards (Good, Fair, and Poor)	
	Representativeness of the Exposed Cohort	Selection of the Non-exposed Cohort	Ascertainment of Exposure	Demonstration that Outcome of Interest Was not Present at Start of Study	Comparability of Cohorts Based on the Design or Analysis Controlled for Confounders	Assessment of Outcome	Was Follow-Up Long Enough for Outcomes to Occur	Adequacy of Follow-Up for Cohorts	
Zafir 2013	*	*	*	*		*	*	*	Poor
Poms 2013	*	*	*	*		*	*		Poor
Strange 2018	*	*	*	*	*	*	*		Fair
Weatherald 2018	*	*	*	*	*	*	*	*	Good
Frank 2020	*	*	*	*	*	*	*	*	Good
Min 2020	*	*	*	*	*	*	*	*	Good

Quality assessment of the included cohort studies using the Newcastle-Ottawa scale. Thresholds for converting the Newcastle-Ottawa scares to AHRQ standards (good, fair, and poor). Good quality: 3 or 4 stars in selection domain AND 1 or 2 stars in comparability domain AND 2 or 3 stars in outcome domain. Fair quality: 2 stars in selection domain AND 1 or 2 stars in comparability domain AND 2 or 3 stars in outcome/exposure domain. Poor quality: 0 or 1 starts in selection domain OR 0 stars in comparability domain OR 0 or 1 starts in outcome/exposure domain.

## Data Availability

All relevant data are reported in the study tables and figures.
